# Genetic fusion of CCL11 to antigens enhances antigenicity in nucleic acid vaccines and eradicates tumor mass through optimizing T-cell response

**DOI:** 10.1186/s12943-024-01958-4

**Published:** 2024-03-08

**Authors:** Hailong Qi, Zhongjie Sun, Tianle Gao, Yanling Yao, Yu Wang, Weiwei Li, Xudong Wang, Xiaofang Wang, Defang Liu, Jian-Dong Jiang

**Affiliations:** 1 Institute of Medicinal Biotechnology, Chinese Academy of Medical Science, Beijing, China; 2 Institute of Materia Medica, Chinese Academy of Medical Sciences & Peking Union Medical College, Beijing, China; 3 State Key Laboratory of Elemento-Organic Chemistry, College of Chemistry, Nankai University, Tianjin, 300071 China; 4 Newish Biological R&D Center, Wuxi, China

**Keywords:** CCL11, Chemokines, Molecular adjuvant, Nucleic acid vaccines and CCR3 + cells

## Abstract

Nucleic acid vaccines have shown promising potency and efficacy for cancer treatment with robust and specific T-cell responses. Improving the immunogenicity of delivered antigens helps to extend therapeutic efficacy and reduce dose-dependent toxicity. Here, we systematically evaluated chemokine-fused HPV16 E6/E7 antigen to improve the cellular and humoral immune responses induced by nucleotide vaccines in vivo. We found that fusion with different chemokines shifted the nature of the immune response against the antigens. Although a number of chemokines were able to amplify specific CD8 + T-cell or humoral response alone or simultaneously. CCL11 was identified as the most potent chemokine in improving immunogenicity, promoting specific CD8 + T-cell stemness and generating tumor rejection. Fusing CCL11 with E6/E7 antigen as a therapeutic DNA vaccine significantly improved treatment effectiveness and caused eradication of established large tumors in 92% tumor-bearing mice (*n* = 25). Fusion antigens with CCL11 expanded the TCR diversity of specific T cells and induced the infiltration of activated specific T cells, neutrophils, macrophages and dendritic cells (DCs) into the tumor, which created a comprehensive immune microenvironment lethal to tumor. Combination of the DNA vaccine with anti-CTLA4 treatment further enhanced the therapeutic effect. In addition, CCL11 could also be used for mRNA vaccine design. To summarize, CCL11 might be a potent T cell enhancer against cancer.

**Supplementary Information:**

The online version contains supplementary material available at 10.1186/s12943-024-01958-4.

## Background

Comprising mRNA or DNA antigen precursors, nucleotide vaccines are taken up by host cells, where their nucleotide sequences are translated into antigens intracellularly. Then, the antigens could be degraded by the proteasome in the cytoplasm to generate epitope peptides for MHC-I molecules and to activate cytotoxic responses. The extracellularly secreted antigens are phagocytized by antigen-presenting cells (APCs) to trigger T helper cell or humoral immunity simultaneously. The characteristics of nucleotide vaccines endowed them with the ability not only in prophylactic but also in therapeutic settings to treat diseases such as cancer. Many clinical trials of tumor nucleic acid vaccines are in progress, of which a therapeutic DNA vaccine for the treatment of cervical intraepithelial neoplasia (CIN), targeting the E6 and E7 antigens of the HPV 16 and 18 strains, has shown a positive effect in patients in a phase 2b trial [1]. Besides, an individualized RNA mutanome vaccine successfully immunized melanoma patients, resulting in a good T-cell response and prolonging progression-free survival [2]. The immunogenicity of nucleic acid vaccines is the key to success. Therefore, further improvement is highly desirable.

Improvement of immunogenicity can be achieved by nucleotide sequence optimization to increase mRNA stability and the expression of antigens [3]. Epitope optimization could promote cross-recognition of wild-type antigens and break immune tolerance to wild-type antigens [4]. Introducing MHC class I trafficking signals (MITD) or lysosomal / endosomal localization signals to assist the process of MHC class I or class II epitope presentation by dendritic cells (DCs) has also been applied to increase cellular immune responses [5, 6]. Gene fusion to combine tetanus toxoid fragments with antigens has been widely used in both DNA and mRNA vaccine design, aiming at strengthening the immune response [7, 8]. However, this approach may lead to a more potent tetanus toxoid fragment response since antigen dominance hierarchies shape CD8 T-cell phenotypes [9]. As antigen expression of the nucleic acid vaccine is extremely low, selective targeting of antigens to APCs is the key to increase effective biological distribution and immunogenicity. APCs have specific receptors that could be targeted by receptor-specific monoclonal antibodies (mAbs) or natural ligands [10]. Delivering antigens together with the interaction between natural ligands such as chemokines and specific receptors on the surface of APCs could realize APC activation, with the advantage of no or weak induction of immune responses of the chemokines themselves and potential adjuvant effects.

Chemokines and chemokine receptors contribute to leukocyte trafficking and recruitment to sites of inflammation. Indeed, XCL1, CCL3 (MIP1α), CCL5 (RANTES), CCL7, CCL20, CCL21, CCL22, CCL25, CCL27, CCL28, CXCL10 and CXCL13 have been demonstrated to significantly promote cellular and humoral responses when fused or co-delivered with antigens from viruses or tumors [11–17]. However, systematic evaluation of the immune response of chemokines after fusing with antigens has not been reported. It remains unclear whether chemokines have different preferences for inducing cellular and humoral immunity. Thus, understanding the full landscape of the immune response shaped by chemokine-fused antigens will provide useful information for the development of chemokine-based preventive or therapeutic vaccines against viruses or tumors and promote the understanding of the coordination of different immune cell subpopulations.

In the present study, we used our DNA tech platform to individually fuse chemokines with the HPV16 E6 and E7 antigens and evaluated the cellular and humoral immune responses in mice after immunization. CCL11 was found to induce the strongest cellular immune response and displayed superior antitumor activity. Different from previous chemokine-fused DNA vaccines, a single immunization of 25 µg CCL11-E6E7 DNA vaccine removed large established tumor masses in multiple experiments, achieving an average tumor clearance rate of over 90%. Furthermore, this technique is fully applicable to mRNA vaccine platforms. Chemokines that are suitable for inducing humoral immune responses were also identified. Our results provided an important foundation for the use of chemokines in vaccine design.

## Methods

### Mice and in vivo electroporation

Eight-week-old C57BL/6N female mice were purchased from Charles River Laboratories Co., Ltd. and raised at Peking Union-Genius Pharmaceutical Technology Company (Beijing, China). The entire experimental process received approval and supervision from the Peking Union-Genius Institutional Animal Care and Use Committees (IACUC). The ethic number is JY23001. Mice were ordered at least one week before the experiment to adapt to the new environment. The mice were first injected with plasmid into the tibialis anterior (TA) muscle followed by an electroporation with 60 V voltage, 10 Hz frequency and 50 ms interval (TERESA).

### Preparation of single cell suspension and flow cytometry analysis

The collected anticoagulated mouse peripheral blood was lysed the red blood cells by adding 1 × red blood cell (RBC) lysis buffer (Biolegend, cat. 420,301). The prepared peripheral blood mononuclear cells (PBMCs) were then subjected to stain and flow cytometric analysis. Single cell suspensions of the spleen and lymph nodes were prepared by direct grinding with a 100 µm cell sieve (Corning). For tumor tissues, they were minced into small pieces and then tissue dissociation solution was added according to the manufacturer's protocol (Miltenyi, cat. 130–095-929). Red blood cell lysis with 1 × RBC lysis buffer was required for the processed spleen. The presence or absence of red blood cells in the lymph node and tumor single cell suspension determines whether red blood cells were lysed. The processed single cell suspension was subjected to staining and analysis by flow cytometry. For the staining of intracellular transcription factors, the BD Fixation/Permeabilization Kit (cat. 554,715) was used. The staining scheme used to distinguish each subpopulation in this article was as follows: E7_49-57_ specific CD8 + T cells (E7_49-57_ tetramer + , CD8 +), MDSC (CD11b + Gr-1 + Ly-6G +), NK cells (CD45 + CD3 − NK1.1 +), CD8 + T cells (CD45 + CD8 +), CD4 + T cells (CD45 + CD4 +), Treg (CD45 + CD4 + Foxp3 +), M1 macrophages (CD45 + CD11b + F4/80 + CD206-), M2 macrophages (CD45 + CD11b + F4/80 + CD206 +). Flow cytometric data was acquired on a BD FACS Canto II flow cytometer (BD Biosciences) and analyzed with FlowJo 7.6.5 software.

### Cell culture and subcutaneous transplantation tumor model construction

The 293 T (cat. CTCC-001–0188), TC-1 (cat. CTCC-400–0328), and B16-OVA (cat. CTCC-007–0623) cell lines were purchased from Zhejiang Meisen Cell Technology Co., Ltd. The 293 T and B16-OVA cells were cultured in DMEM medium supplemented with 10% fetal calf serum (FBS). TC-1 cells were cultured in RPMI1640 medium supplemented with 10% FBS. When the cells reached the logarithmic growth phase, TC-1 and B16-OVA cells were digested with trypsin into single-cell suspensions, and the cell concentration was adjusted to 2 × 10^5^ per 100 µl and inoculated subcutaneously into mice.

### Western blot assay

Twenty-four hours after the transfection of the plasmid into the 293 T cells, the cells were harvested and lysed with RIPA buffer (50 mM Tris–HCl, pH 8.0, 150 mM NaCl, 1.5 mM MgCl_2_, 0.1% SDS, 0.5% deoxycholate (DOC), 1% NP-40) supplemented with 1 mM PMSF, 1 × protease inhibitor mixture (Roche). After centrifugation at 12,000 rpm for 10 min at 4 °C, the supernatant was collected and SDS-PAGE loading buffer (5 ×) was added. The samples were boiled for ten minutes following a by separation on 10%SDS-PAGE gels. Then the samples were blotted using the anti-FLAG antibody (Sigma, #1804).

### Cell depletion

After grouping mice according to tumor size, CD4 (BioXcell, cat. BE0119. 350 µg per mouse), CD8 (BioXcell, cat. BP0061. 350 µg per mouse), NK1.1 (BioXcell, cat. BE0036. 250 µg per mouse), and CCR3(BioXcell, cat. BE0316. 250 µg per mouse) blocking antibodies were administered intraperitoneally every three days for a total of seven times. The cell depletion effect was detected by FACS after the third administration.

### mRNA preparation and formulation

Synthetic DNA fragments encoding the protein of interest were cloned into Genscript plasmid vectors containing sequences corresponding to the T7 promoter, a 5' untranslated region (UTR), a 3' UTR, and a 31 + 10 nt spacer + 71 nucleotide poly A tail. The maps and sequences are included in the supplementary materials. Quality control passed plasmids were linearized with the class -IIS restriction enzyme BspQI to generate a template with no additional nucleotides beyond poly A. Linearized plasmid DNA purified by ethanol precipitation was subjected to in vitro transcription (IVT) with T7 RNA polymerase (Genscript). In the presence of 10 mM N1-methylpsedouridine-5'-triphosphate, adenosine 5'-triphosphate, cytidine 5'-triphosphate and guanosine 5'-triphosphate. This IVT process also included a co-transcriptional capping reagent capable of forming a cap1 structure. The RNA was further purified using NGS magnetic beads. The RNA concentration and quality were evaluated by spectrophotometry and capillary gel electrophoresis systems. The preparation of the liposomes and the formation of lipoplex (LPX) were performed according to a previous study described [18].

### Protein purification

The GST-tagged HPV16-E6E7 sequence was cloned into pGEX-6P-1 vector and transfected into E. coli BL21 (DE3) strain for expression under a final concentration of 500 μM Isopropyl-β-D-thiogalactopyranoside (IPTG) inducing at 16 °C for 18 h. Cells were then harvested by centrifugation at 6500 g for 15 min and suspended in PBS buffer for cell disruption using low temperature ultra-high pressure continuous flow cell disrupters (ATSHPH AH-NANO). The supernatant of cell lysis was collected by centrifugation at 11000 g for 60 min, followed by a one-step affinity chromatography using GST-tag purification resin (BeyoGold, Lot No. P2253) for purifying GST-tagged proteins.

### ELISA

Five micrograms of GST-tagged HPV16-E6E7 protein suspended in PBS buffer was coated on the Corning ELISA plates at 4 °C overnight. Mouse plasma was obtained at 21 days after immunization. The serum was separated and incubated with the plates after blocking with 1 × ELISAPOT Diluent (Invitrogen, cat. 00–4202-56) for 2 h at room temperature (RT) with a 1:100 dilution overnight at 4 °C. After five times washings, the goat anti-mouse IgG2a-HRP (Southern Biotech, cat. 1081–05) were added into the plates with a 1:5000 dilution for 1 h at RT. Finally, after washing the plates, TMB 1-Component Peroxidase Substrate (Invitrogen, cat. 00–4201-56) was used to indicate the reaction which was stopped using a 2 M HCl solution. The absorbance at 450 nm was determined within 30 min using a Synergy HTX instrument (BioTek Instruments, Highland Park, VT).

### RNA-Seq Library Construction and Sequencing

Total RNA (1 µg) from each treated or control group was used to enrich poly(A) mRNA using oligo(dT) magnetic beads (Invitrogen, USA). RNA-seq libraries were then prepared using the NEBNext® UltraTM RNA Library Prep Kit for Illumina® (NEB, USA) according to Illumina's library construction protocol. The libraries were sequenced to a depth of 20 million reads on the Illumina Novaseq platform. RNA library construction and next-generation sequencing were performed at Novogene. Raw reads were generated and sorted by index codes for further analysis. Low quality and adaptor sequences were trimmed using Trimmomatic v 0.39 software to obtain clean data for downstream analyses.

### Gene expression analysis

Clean reads from each library were mapped to the reference genome using Hisat2 v2.2.1. FeatureCounts v1.5.0 was then used to count the number of reads mapped to each gene. Genes with less than 10 mapped reads in the total sample were excluded. Samples were analyzed by DESeq2 to obtain log2 fold change and corresponding p-value in R v4.2.0. Differentially expressed genes were identified from these transformed values using the criteria of log2 (fold change) > 0 and p adjustment value < 0.05. The Benjamini–Hochberg method was used to adjust p-values to control for false discovery rate. R studio was used to run custom R scripts to perform principal component analysis (PCA), volcano plots, and heat maps.

### Enrichment analysis of differentially expressed genes

Gene Ontology (GO) enrichment analysis of differentially expressed genes was performed using the cluster Profiler R package. GO terms with a Bonferroni adjusted p-value of less than 0.05 were considered significantly enriched. R studio was used to execute custom R scripts to perform the bubble plot.

### TCR clonality analysis

T cell receptor clonality was assessed from the RNAseq data using the MiXCR v3.0 tool. MiXCR applied the standard parameters described in the RNAseq workflow manual to obtain clonotypes from the raw fastq files. After obtaining the quantified clonotypes, the R package immunarch V1.0 was used to calculate sample diversity and counts, respectively. The diversity of T cell receptor clonotypes was determined using the chao1 index, which is a nonparametric asymptotic estimator of species richness.

### Statistics

Statistical analyses, unless otherwise indicated, were performed using GraphPad Prism 5. P values below 0.05 were considered statistically significant. Data are shown as the mean ± SEM.

## Results

### Identification of CCL11 for fusion with antigens to improve immunogenicity and in vivo antitumor activity

**Fig. 1:**
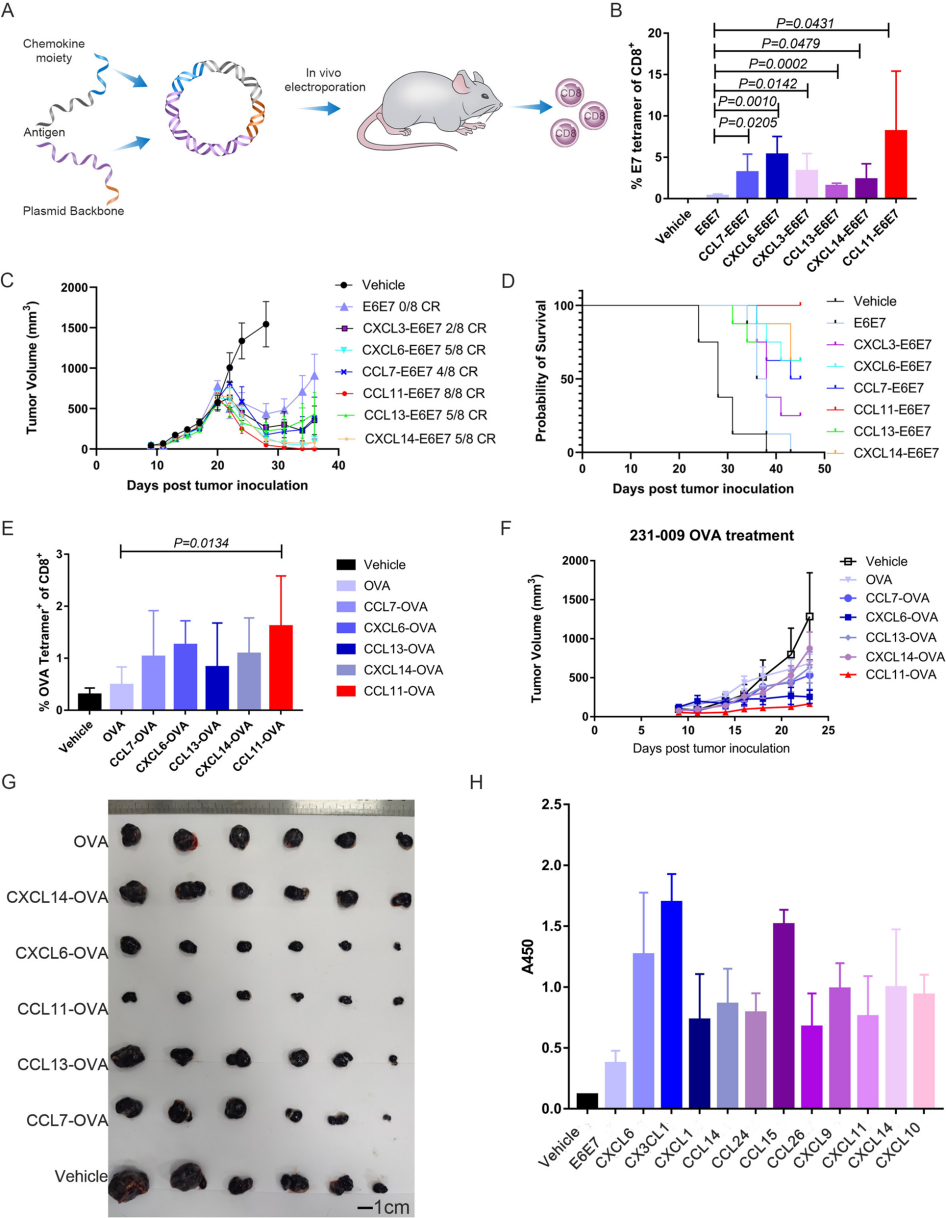
Screening and identification of chemokines for fusion with antigens to improve immunogenicity. **A** Schematic diagram of the cellular immune screening process. Chemokines and E6E7 antigens were assembled into plasmid DNA and immunized animals for evaluation. **B**-**D** Verifying the cellular immune response and anti-tumor effect of the six selected chemokines. 2 × 10^5^ TC-1 cells were subcutaneously transplanted, and vaccine injection was initiated at an average 50mm^3^ tumor volume. **B** Fourteen days after the vaccination, specific T cell levels in peripheral blood were detected by flow cytometry. Bar graphs (mean ± SEM, *n* = 5) show the percentage of E7_RAHYNIVTF_ tetramer + of CD8 + T cells. **C** The tumor size is measured every other day and plotted as a tumor growth curve. Mean volume ± SEM was used to represent the average tumor size for each group of mice (*n* = 8). The complete remission ratio was indicated. **D** The survival status of the mice is recorded and plotted as a survival curve. If the tumor volume exceeds 2000mm^3^ or continues to grow for a week after shrinking, it is considered dead. **E** Vaccines of different chemokines were administrated 3 days post 5 × 10^5^ B16-OVA cells were subcutaneously transplanted. Bar graphs (mean ± SEM, *n* = 6) show the percentage of OVA tetramer + of CD8 + T cells from blood 14 days post vaccine immunization. Statistical significance was calculated between CCL11-OVA and OVA groups using a student's *t*-test. **F** Average growth curve of implanted B16-OVA tumors in C57BL/6 mice was shown with treatment of different fusion plasmids. Mean volume ± SEM was used to represent the average tumor size for each group of mice (*n* = 6). **G** When the tumor volume in the vehicle group grew to an average value of 1400 mm.^3^, all mice from each group were euthanized. Pictures showed the tumors dissected from OVA, CXCL14-OVA, CXCL6-OVA, CCL11-OVA, CCL13-OVA, CCL7-OVA, and Vehicle from top to bottom. The tumors were arranged in order of size. Scale bar: 1 cm (*n* = 6). **H** Five micrograms of GST-tagged HPV16-E6E7 protein suspended in PBS buffer was coated on the Corning ELISA plates. The mouse serum obtained at 21 days after immunization was analysed with a 1:100 dilution. Bar graphs (mean ± SEM, *n* = 5) show the ELISA reactivity of IgG2c in the serum. Result is expressed as the optical densities at 450 nm (A450)

To systematically evaluate the immunomodulatory effect of fused chemokines on antigens, we selected the E6 and E7 proteins of HPV16 as antigens for fusion. The E6 and E7 proteins were assembled according to a previous study and are hereafter referred to as E6E7 in this article [19]. We then added the chemokine sequence to the N terminus of E6E7 linked by a flexible G5SG5 linker which is rich in hydrophilic amino acids and increases spatial separation between two domains allowing for proper folding to preserve optimal biological activity of chemokines [20]. Since DNA vaccines are administered by intramuscular injection and are mainly expressed at the injection site, the secretion signal peptide of the fused chemokines is retained to achieve secretion expression for APC targeting. Most of the chemokines were mouse derived, while for some of the chemokines, we used human surrogates in the absence of mouse information. The fused sequences were cloned into the pVAX 1 plasmid vector and transfected into HEK 293 T cells to detect protein expression. The general process of screening is shown in Fig. 1A. We synthesized a total of 46 chemokines and FMS-like tyrosine kinase 3 ligand (Flt-3L). Due to the different synthesis rates of different chemokine-fused E6E7 constructs and the need for further optimization of the sequences of some non-expressed fusion proteins after synthesis, we obtained plasmids and immunized animals in several batches. Animals were immunized separately with a 25 µg dose of chemokine-fused plasmid and we used 50 µg E6E7 plasmid as a control in every vaccination to screen a true enhancement of chemokines. Due to the differences in the intensity of the T-cell immune response induced by the same plasmid during each electroporation, we normalized the specific T-cell immune response values from different batches using the average specific CD8 + T-cell value of E6E7 plasmid immunized mice each time. A change in the mean CD8 + -specific T-cell immune response greater than or equal to twice that of the E6E7 group was set as the screening threshold (Fig. S1A). Based on the protein expression levels (Fig. S1B), we tentatively identified that six chemokines, including CCL11, had immune-enhancing effects (Fig. S1A and B). We re-evaluated the immunogenicity and antitumor activity of the six chemokines in TC-1 tumor-bearing mice. All screened chemokines retained the improvement in immunogenicity compared to the E6E7 plasmid, with CCL11 showing the highest specific CD8^+^ T-cell level (Fig. 1B). Tumor growth was inhibited or progressively eradicated in mice in all chemokine-fused E6E7 groups, while treatment with E6E7 plasmid alone only showed short-term growth inhibition followed by recurrence (Fig. 1C). The CCL11-E6E7-treated mice displayed the lowest mean tumor growth rate and a significantly prolonged survival period, with complete remission in 4 out of 5 mice whose therapeutic efficacy could be repeated (Fig. 1D, E, Fig. S1C). To verify whether CCL11 fusion is superior to a simple co-administration, we constructed a plasmid expressing CCL11 and injected CCL11-E6E7, E6E7 and CCL11 simultaneously for comparison with E6E7. Both CCL11-E6E7 and co-administration of CCL11 with E6E7 showed a significant increase of E7-specific CD8^+^ T cells when compared to the levels obtained by E6E7 treatment. However, CCL11-E6E7 still had the highest T-cell level indicating that fusion with CCL11 favors the induction of specific CD8^+^ T cells (Fig. S1D). Our results are consistent with the findings of previous studies, which demonstrated that the fusion of CCL19 and XCL1 was more advantageous than the co-administration [21, 22]. This enhanced immunogenicity and antitumor activity of CCL11 was preserved in B16-OVA tumor-bearing mice treated with the identified chemokine fused to the ovalbumin (OVA) antigen (Fig. 1F, G). These results indicated that different chemokine-fused antigens differ in their ability to induce cellular immunity and corresponding antitumor effects.

Immune editing mediated by the selective pressure of the immune system on tumor cells may promote cancer antigenic heterogeneity and immune evasion. We also tested whether vaccination with antigen fused with CCL11 eliminated tumor cells without antigen expression in a prophylactic model. After 14 days of CCL11-OVA and control OVA plasmid vaccination, B16-OVA and B16 tumor cells were sequentially inoculated. CCL11-OVA prevented the formation of any palpable tumors from either cell lines, indicating that a multiple antigenic response was established to preclude the development of an antigenically heterogeneous tumor (Fig. S1E). In addition to the specific T-cell immune response, we also preliminarily evaluated chemokine fusion-mediated humoral immune responses. By setting the same threshold employed to assess the cellular immune response, the enhanced humoral immune responses generated by the chemokines were confirmed. CX3CL1 and CXCL6 maximized the humoral immune response. The chemokines that improved the humoral immune response did not fully overlap with those that enhanced the cellular immune response (Fig. 1B and H and Table S1).

### Enhancement of antigenicity with CCL11 fusion for application in mRNA vaccines

We then tested whether CCL11 could also increase the immunogenicity of antigens in the mRNA or self-amplification mRNA (saRNA) vaccine platform. CCL11-E6E7 and E6E7 mRNA were synthesized and encapsulated into lipid nanoparticle (LNP) carriers as previously described [23]. CCL11-E6E7 mRNA showed the same level of protein expression as E6E7 mRNA (Fig. S2A). Next, 10 µg of each type of mRNA was injected into the skeletal muscle of TC-1 tumor-bearing mice. Specific CD8^+^ T-cell detection showed that CCL11-E6E7-treated mice had approximately twice the level of CD8^+^ T cells as the E6E7 group, which was correlated with long-term disease remission in 5/6 mice, while only 1/6 mice in the E6E7 group achieved complete remission (Fig. S2B, C). Survival analysis also showed significant benefits in the CCL11-E6E7-treated group (Fig. S2D). Generally, saRNA vaccines can induce a stronger immune response than mRNA vaccines due to more efficient antigen expression (Fig. S2A). To obtain a clearer therapeutic effect of saRNA, we chose larger established mouse tumors with an average volume greater than 100mm^3^ and treated them with a single dose of 2 µg saRNA. Compared to E6E7 saRNA, CCL11-E6E7 saRNA showed slightly lower protein expression (Fig. S2A). However, CCL11-E6E7 saRNA showcased stronger specific T cell immune response (Fig. S2E). Although significant overall survival benefits have not been statistically determined, the CCL11-E6E7 saRNA treated group displayed better tumor treatment efficacy, achieving durable complete remission on 7/7 mice (Fig. S2F and G). Only 4/7 mice displayed durable complete remission of the E6E7 saRNA group (Fig. S2F and G). These results suggest that the chemokine fusion strategy is suitable for both DNA and mRNA vaccine preparation.

### CCL11-E6E7 vaccination promoted both specific CD8 + T cells and infiltration of innate immune cells into the tumor microenvironment

**Fig. 2:**
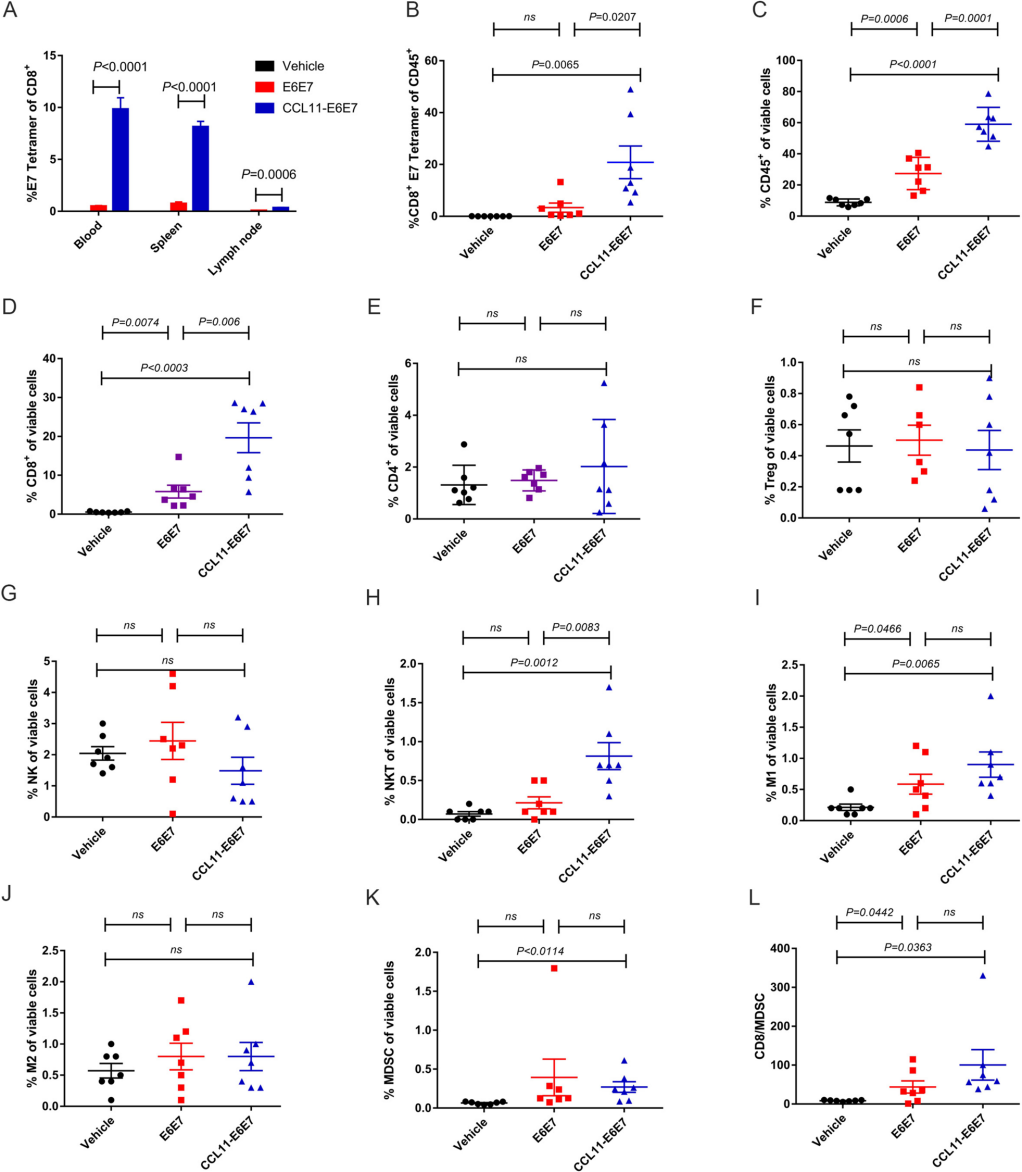
Comparison of the proportions of adaptive and innate immune cells in TC-1 tumor bearing mice induced by plasmid vector (vehicle), E6E7 and CCL11-E6E7 vaccination for once (*n* = 7 for each group). **A** Bar graphs (mean ± SEM) show the percentage of E7 tetramer + cells of CD8 + T cells from blood, spleen and lymph node in each group. **B**-**K** The tumor was removed and digested into single cell suspension for FACS analysis of adaptive and innate immune cells. Scatter plots (mean ± SEM) show the percentage of (**B**) CD8 + E7 tetramer + of CD45 + cells. **C**-**K** Scatter plots (mean ± SEM) show the percentage of (**C**) CD45 + cells, (**D**) CD8 + cells, (**E**) CD4 + cells of viable cells (**F**) Treg cells, (**G**) NK cells, (**H**) NKT cells, (**I**) M1 macrophage, (**J**) M2 macrophage, (**K**) MDSC cells and. **L**, Scatter plots (mean ± SEM) show the ratio of CD8/MDSC cells. Statistical significance was calculated using a student's t-test, where "ns" stands for not significant

To understand why fusion with CCL11 enhanced antitumor activity, specific CD8 + T cells in the peripheral blood, secondary lymphoid tissues, and tumors of TC-1 tumor-bearing mice were analyzed 14 days after CCL11-E6E7 or E6E7 immunization using flow cytometry. Consistent with the results in Fig. 1A, immunization with 25 µg CCL11-E6E7 significantly increased E7-specific CD8 + cytotoxic lymphocytes in the peripheral blood, spleen, and lymph nodes, and even within tumors, exhibiting the lowest proportion of lymphocytes in lymph nodes and the highest proportion in tumors (Fig. 2A and B). Tumor-infiltrating immune cell subpopulations were also analyzed. The tumors treated with CCL11-E6E7 showed a significant increase in total leukocyte infiltration, with leukocytes accounting for more than half of the viable cells (Fig. 2C). We then analyzed adaptive immune cells and innate immune cells. The proportion of CD8 + T cells was significantly increased in CCL11-E6E7-treated tumors, with no significant difference in CD4 + T cells (Fig. 2D and E). There was also no difference in Treg cells, an immunosuppressive population of CD4 + T cells (Fig. 2F). Although the proportion of NK cells did not change significantly, NKT cells showed significant upregulation after CCL11-E6E7 vaccination (Fig. 2G and H). The frequency of macrophages polarized toward the proinflammatory M1 subtype was increased, while the frequency of M2 macrophages, which have inhibitory antitumor effects, remained unchanged in the three groups (Fig. 2I and J). Myeloid-derived suppressor cells (MDSCs) were also increased in the CCL11-E6E7 treatment group compared to the vehicle group, but the ratio of CD8 + T cells to MDSCs was significantly higher in the CCL11-E6E7 treatment group than in the other groups (Fig. 2K and L). Overall, the fusion of CCL11 enhanced the enrichment of cell populations with antitumor immune functions in tumors.

### CCL11-E6E7 immunization induced the activation of both innate and adaptive immune gene signatures in tumors

**Fig. 3:**
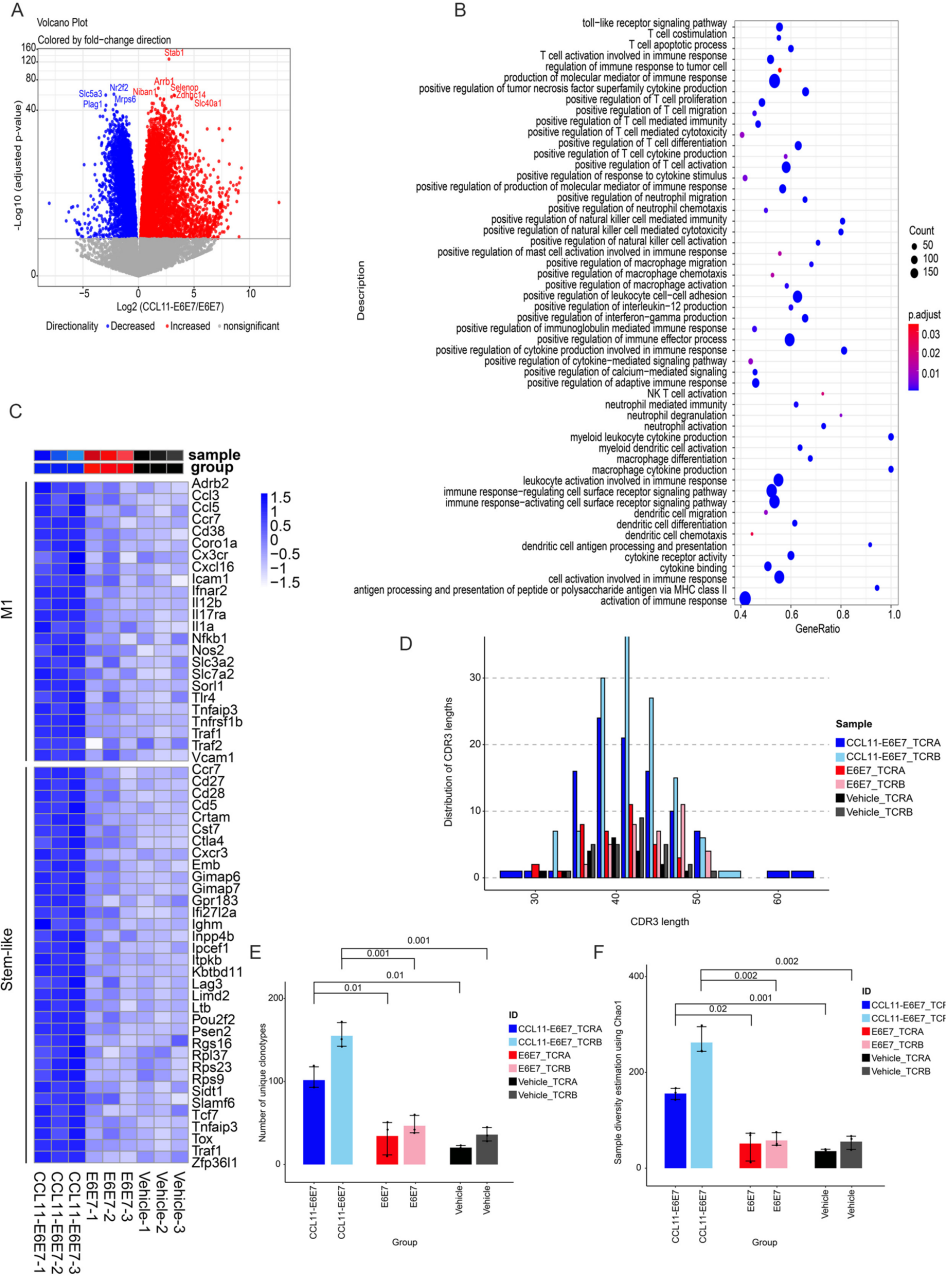
RNA-seq transcriptome analysis identified the differentially expressed genes in tumor treated with CCL11-E6E7, E6E7 or vehicle. After the grafted TC-1 tumor is palpable, one dose of 25 µg CCL11-E6E7, E6E7, or an empty vector was intramuscular injection followed by electroporation. The tumor was removed and subjected to RNA-seq analysis on the fourteenth day. **A** Volcano plot was used to visualize the DEGs in tumor tissue immunized with CCL11-E6E7 compared to E6E7. The red dots represent significantly up-regulated genes and the blue dots represent significantly down-regulated genes as calculated by |log2 FC|> 0 and FDR < 0.05. **B** The GO enrichment analysis was performed on genes that were significantly up-regulated by CCL11-E6E7 vaccination. Enriched pathways were determined with a significance cutoff of *p*-value < 0.05 (Fisher’s exact test) and a requirement for at least 40% up-regulated genes in the pathway. **C** The upregulated DEGs extracted from CCL11-E6E7 vs E6E7 that are positive associated with M1 macrophages and stem-like T cell activation in the tumor tissue were used to perform hierarchical clustering. **D** TCR complementarity-determining region 3 (CDR3) length distribution patterns among TCRA and TCRB reads in each group. The x-axis represents the CDR3 length in nucleotide (nt) and the y-axis represents the number of each length group in all the identified CDR3 sequences. **E**, **F** The T cell receptor (TCR) clonotypes showed increased abundance and expansion upon CCL11-E6E7 treatment compared to E6E7 treatment. The TCRA and TCRB sequence analysis was used to quantify the number of TCR clonotypes detected in each treatment group (*n* = 3/group). The chao1 indexes were calculated as a measurement for TCRA and TCRB clonal diversity. The p-value was measured by Student's t-test

To fully understand the immune status inside the tumors treated with CCL11-E6E7, we extracted total RNA and performed RNA-seq analysis of the tumors on day 14 after immunization. Principal component analysis showed that the E6E7 treatment group was similar to the empty vector group, while the CCL11-E6E7 treatment group was completely separated from them, which was consistent with the results of the clustering analysis of all differentially expressed genes (DEGs) (Fig. S3A and B and Table S2). We mainly analyzed the DEGs of tumors treated with CCL11-E6E7 compared to E6E7. A total of 3381 genes were upregulated and 1836 genes were downregulated after CCL11-E6E7 vaccination (Fig. 3A). Gene Ontology (GO) pathway enrichment analysis of the upregulated genes revealed enrichment of both innate and adaptive immune pathways, with a cutoff of at least 40% of genes upregulated in a related pathway (Fig. 3B). Compared to the E6E7-treated tumors, CCL11-E6E7-treated tumors showed many upregulated genes that were found to be enriched in the positive regulation of both innate and adaptive immune pathways. In addition to T-cell activation-related pathways, we also observed that NK cells, NKT cells, dendritic cells (DC), neutrophils, and macrophages migration and activation pathways were enriched which is consistent with the results of intratumoral immune subpopulations analysis in Fig. 2 (Fig. 3B). CCL11-E6E7 treatment induced comprehensive immune activation within the tumor. As M1 polarization of macrophages promotes antitumor activity and stem-like T cells are mainly considered to have antitumor activity and are believed to be the cells responsible for response to immune checkpoint therapy, we further mapped the genes correlated with M1 subtype polarization of macrophages and stem-like T cells [24, 25]. Genes related to the M1 phenotype and stem-like T cells, such as *cd38* and *tcf7*, were upregulated in the CCL11-E6E7-treated tumors, supporting the presence of differentiation of macrophages and T cells toward an antitumor state (Fig. 3C).

### CCL11-E6E7 immunization promoted intratumoral TCR repertoire diversity

The results of RNA-seq revealed the activation of numerous T-cell immune response pathways, and the increased diversity of the cytotoxic T-lymphocyte repertoire has been shown to be associated with the generation of high-avidity, protective T cells [26]. We therefore analyzed TCR repertoire diversity using a previously described method for extracting TCR information from bulk RNA sequencing data [27]. Since the CDR3 loops are hypervariable, representing junctional diversity, we first assessed the distribution of TCRα and TCRβ CDR3 reads of all detected lengths in the three groups of tumors. CCL11-E6E7-treated tumors showed significantly more variety in CDR3 length (Fig. 3D). Moreover, CCL11-E6E7 therapy indeed led to a diversification of the T-cell repertoire within tumors, as evidenced by the detection of unique TCRα and TCRβ CDR3 sequences. Compared to E6E7-treated tumors, a significant increase in the total number of clonotypes was observed in CCL11-E6E7-immunized mice (Fig. 3E). We also evaluated the genetic diversity of TCRα and TCRβ using the Chao1 index [28]. The results showed that CCL11-E6E7 immunization caused a significant expansion of gene diversity, with TCR clonotypes showing increased abundance and expansion upon CCL11-E6E7 treatment (Fig. 3F).

### The antitumor effect of CCL11 was dependent on CD8 + T cells, NK cells and CCR3 + cells

**Fig. 4:**
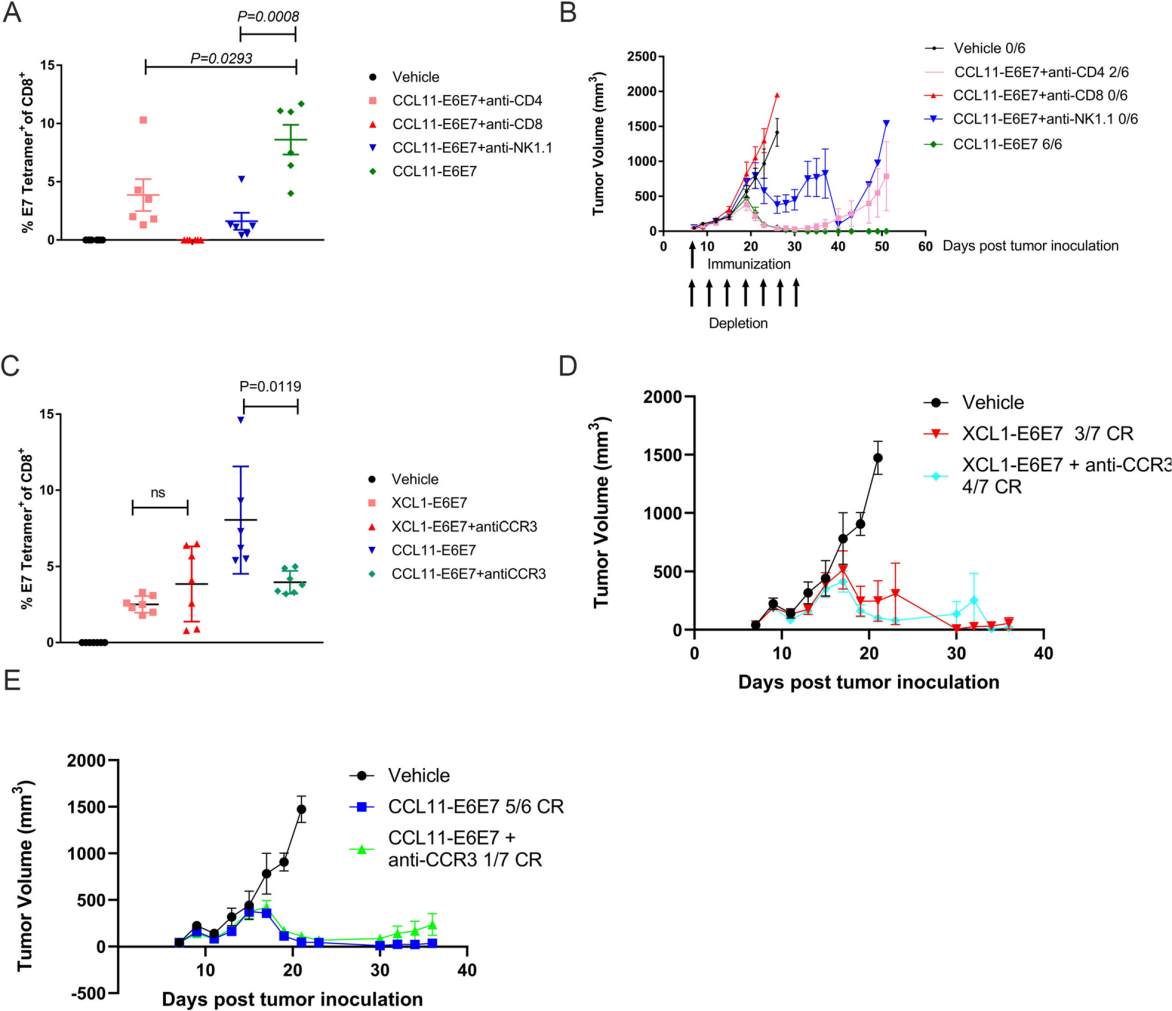
Therapeutic effects of CCL11-E6E7 relies on CD8 + T, NK and CCR3 + cells for durable tumor inhibition. **A** and **B** The mice (*n* = 6 for each group) were each inoculated with 2 × 10^5^ TC-1 tumor cells which express HPV16 E6 and E7 protein. After 7 days, the mice were treated with CCL11-E6E7 plasmid alone or combined with anti-CD4, anti-CD8, or anti-NK 1.1 administration every three days for a total of 7 times. Following treatment, the percentage of E7-specific CD8 + T-cells in the peripheral blood were analyzed by Fluorescence activated cell sorting (FACS) analysis (**A**) and the growth of tumors is regularly measured and plotted as a curve (**B**). Data are presented as means ± SEM. **C** and **D**-**E** On the 7th day after being grafted with 2 × 10.^5^ TC-1 tumor cells subcutaneously, the mice are grouped (*n* = 7 for each group,) according to tumor size and treated with XCL1-E6E7 or CCL11-E6E7 with or without anti-CCR3 monoclonal antibody injection intraperitoneally. FACS analysis on peripheral blood was conducted to evaluate the percentage of E7-specific CD8 + T-cells at 14 days post immunization whose data are presented as means ± SEM (**C**).Kinetics of tumor growth are shown as means ± SEM for each group and the complete remission ratio was indicated (**D**-**E**)

Both adaptive and innate populations were observed in the microenvironment of CCL11-E6E7-treated tumors. We therefore depleted CD4 + T cells, CD8 + T cells, or natural killer (NK) cells to determine which population might be responsible for tumor elimination in mice bearing TC-1 tumors. After three times of monoclonal antibody administration, the CD4 + and CD8 + T cells were almost completely depleted, and NK cells were reduced to approximately 0.1% in peripheral blood (Fig. S3C and D). Only CD8 + T-cell depletion completely abolished the initial tumor regression and late tumor eradication induced by the CCL11-E6E7 vaccination (Fig. 4A). CD4 + T-cell depletion reduced the expansion of specific CD8 + T cells induced by CCL11-E6E7 vaccination, and mice showed comparable initial antitumor responses but were less likely to maintain durable remission than those that received CCL11-E6E7 vaccination (Fig. 4A and B). Interestingly, both the expansion of specific CD8 + T cells and the antitumor responses were compromised by NK cell depletion (Fig. 4A and B). Collectively, these results suggest that CD8 + T and NK cells are necessary for the tumor inhibition and eradication induced by CCL11-E6E7 immunotherapy and CD4 + T-cell help the expansion of specific CD8 + T cells and durable remission.

As CCR3 is the major receptor of CCL11, we sought to determine whether CCR3 + cells were essential for immunogenic improvement and tumor eradication in vivo. We evaluated the immunogenicity and anti-tumor effect of CCL11-E6E7 in mice with or without CCR3 + cells by using an anti-CCR3 monoclonal antibody to deplete CCR3 positive cells. The depletion of CCR3 + cells should not affect the immunogenicity of E6E7 fused with chemokines whose receptor is not CCR3. To demonstrate the on-target effects of anti-CCR3 antibody, we also evaluated the immunogenicity and anti-tumor effect of XCL1-E6E7 with XCR1 as the specific receptor in mice with or without CCR3 + cells [29]. Only 50% depletion of CCR3 + cells was achieved after three doses of anti-CCR3 antibody administration (Fig. S3E). We found that there was indeed no effect on both E7-specific CD8 + T cells (Fig. 4C) and tumor complete remission (CR) after CCR3 + cell depletion in XCL1-E6E7 treated mice bearing TC-1 tumors (3/7 vs 4/7 CR, Fig. 4D). However, CCR3 + cell depletion led to a decrease in E7-specific CD8 + T cells (Fig. 4C) in CCL11-E6E7 treated mice. Though the depletion of CCR3 + cells showed almost no effect on initial tumor regression, almost all mice later succumbed to tumors, specifically in the CCL11-E6E7 group (5/6 vs 1/7 CR, Fig. 4E). These results support the involvement of CCR3 + cells in the function mediated by CCL11, but a more definitive removal of CCR3 + cells is necessary for further verification.

### Vaccination with CCL11-E6E7 generated more TCF1 + PD-1- CD8 + T cells

**Fig. 5:**
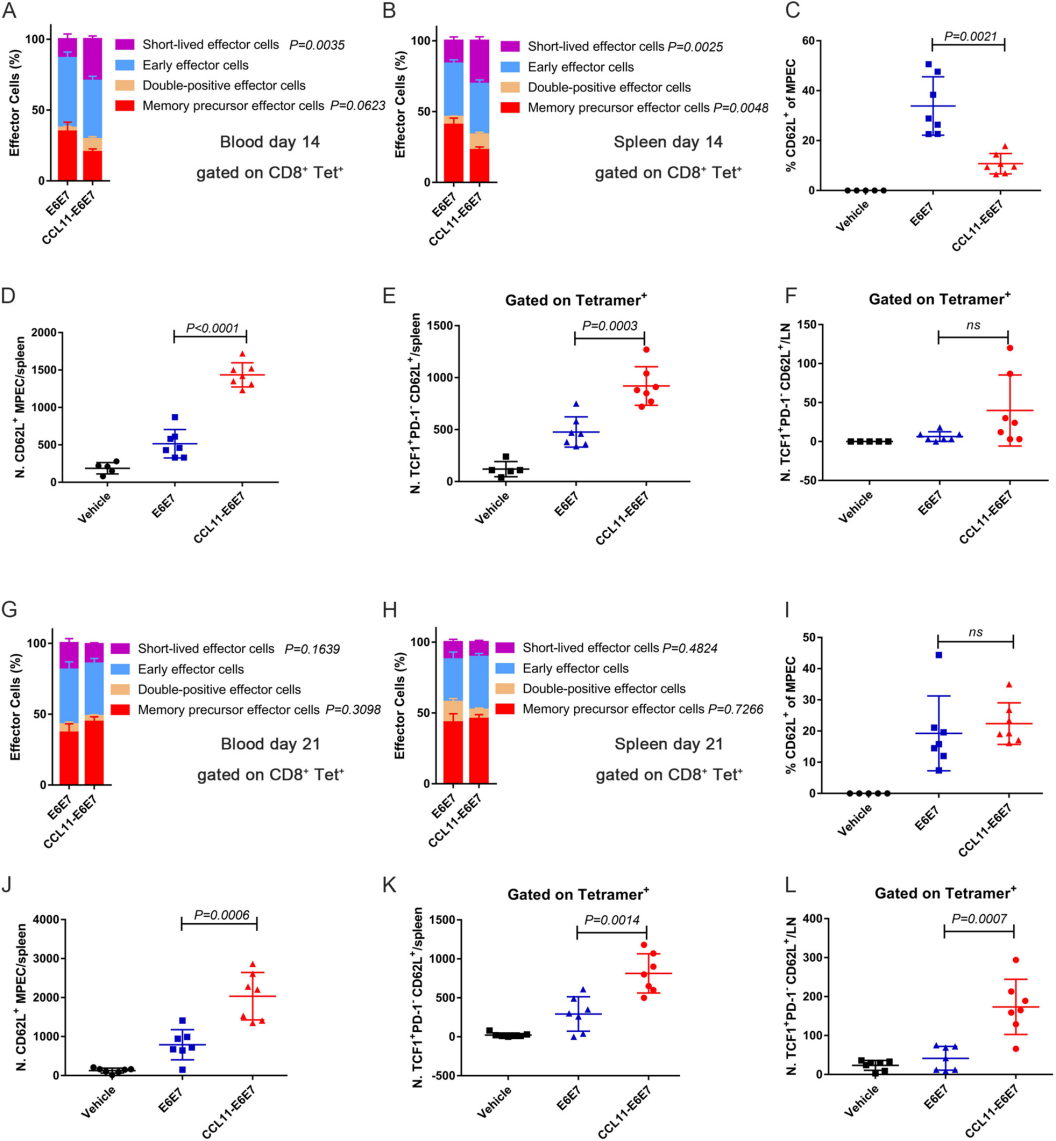
Dynamic analysis of phenotypic differentiation of specific CD8 + T cell in blood, spleen and lymph nodes. **A** and **B** The frequencies of SLEC and MPEC subpopulations of CD8 + tetramer + T cells (mean ± SEM) in blood (**A**) and spleen (**B**) at 14 days after E6E7 or CCL11-E6E7 immunization (*n* = 7 mice for each group). P-value reflects statistical differences in specified subgroups. **C** Graphs (mean ± SEM) show the percentage of CD62L + T cells of MPEC cells in spleen on day 14. **D** Graphs (mean ± SEM) show the number of CD62L + MPEC cells in spleen on day 14. **E** and **F** Graphs (mean ± SEM) show the number of TCF1 + PD-1- CD62L + cells of tetramer + cells in the spleen (**E**) or lymph node (**F**) on day 14. **G** and **H** The frequencies of SLEC and MPEC subpopulations of CD8 + tetramer + T cells (mean ± SEM) in blood (**G**) and spleen (**H**) at 21 days after E6E7 or CCL11-E6E7 immunization (*n* = 7 mice for each group). *P*-value reflects statistical differences in specified subgroups. **I** Graphs (mean ± SEM) show the percentage of CD62L + T cells of MPEC cells in spleen on day 21. **J** Graphs (mean ± SEM) show the number of CD62L + MPEC cells in spleen on day 21. **K** and **L** Graphs (mean ± SEM) show the number of TCF1 + PD-1- CD62L + cells of tetramer + cells in the spleen (**E**) or lymph node (**F**) on day 21. Statistical significance was calculated using a student's t-test, where "ns" stands for not significant

The formation of high-magnitude and -quality immunological memory of specific T cells is crucial for the long-term efficacy of vaccines against infectious diseases or tumors. Fusion with CCL11 increased the magnitude of specific T cells and antitumor efficacy, as shown in Figs. 1 and 2. We next tested whether it affected the immunological memory and stemness of T cells. Changes in CD8 + memory-predictive effector cells (MPECs) or short-lived effector cells (SLECs) over time were dynamically evaluated using the expression of CD127 and KLRG1 as markers. CCL11-E6E7 immunization produced a higher proportion of SLECs, while E6E7 immunization produced a higher proportion of MPECs in the peripheral blood and spleen on day 14 (Fig. 5A and B). Analysis of the proportion of central memory T (Tcm) cells showed that the E6E7 group had a higher proportion of Tcm cells in MPECs (Fig. 5C). However, the total number of Tcm cells in the spleen of the CCL11-E6E7 group was significantly higher than that of the E6E7 group (Fig. 5D). A recent study identified TCF1 + PD1- stem-like T cells in the lymph nodes as the main cell population responsible for antiviral and antitumor effects [30]. We found the total number of TCF1 + PD1- stem-like T cells was also higher in the CCL11-E6E7 group than that in the E6E7 group in the spleen but not in the lymph nodes (Fig. 5E and F). However, on day 21 after CCL11-E6E7 immunization, the proportions of MPECs and Tcm cells among MPECs in the blood and spleen were not different or were even better than those in the E6E7 group, with the number of Tcm cells in the spleen still higher (Fig. 5G-J). In addition, the total count of TCF1 + PD1- stem-like T cells was also higher in the CCL11-E6E7 group than in the E6E7 group in both the spleen and lymph nodes (Fig. 5K and L). These data suggested that CCL11 fusion with antigens promoted CD8 + T-cell stemness, early effector cell differentiation and late memory formation.

### Combination with a CTLA-4 inhibitor eradicates established HPV + tumors

**Fig. 6:**
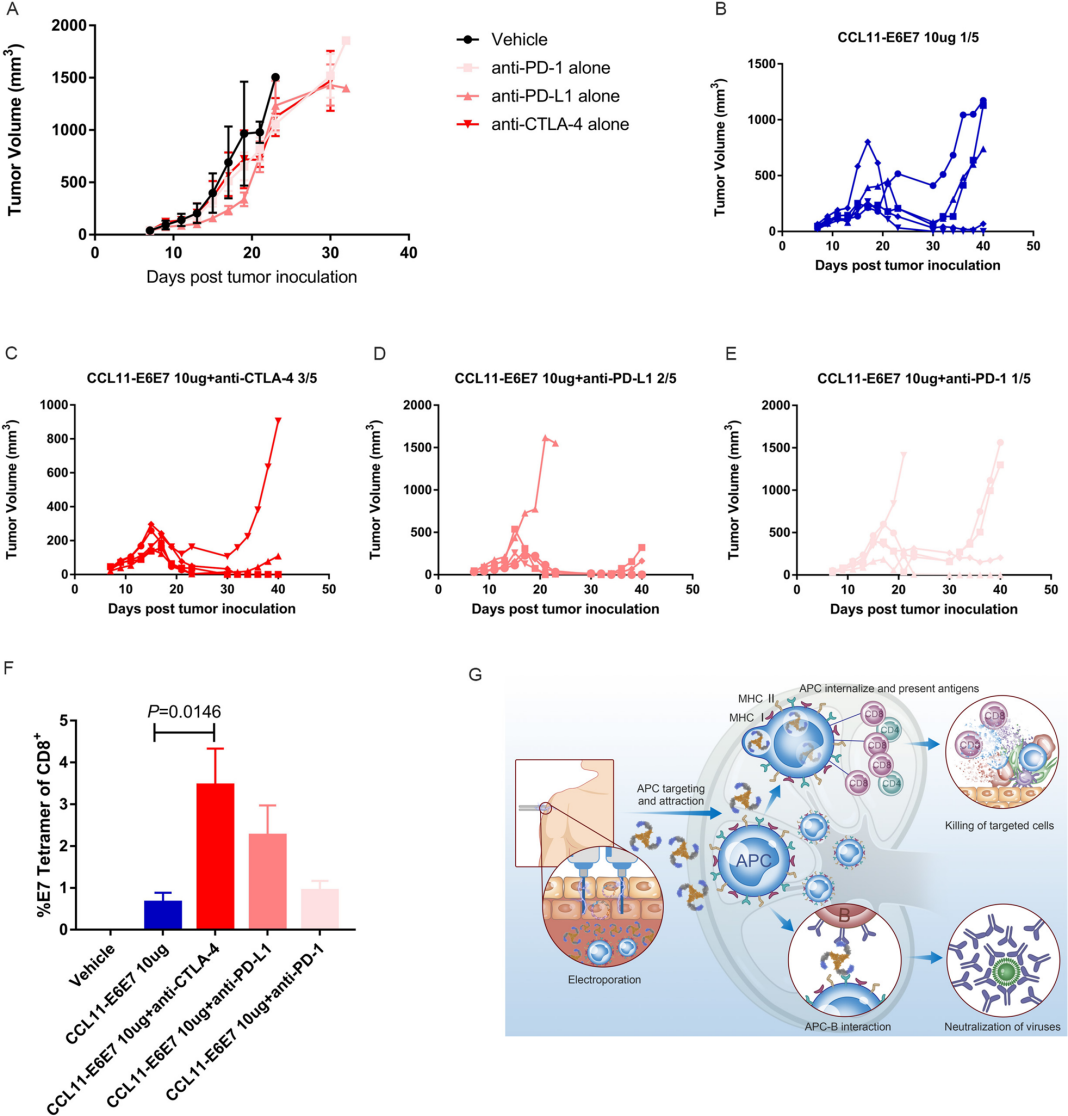
Combination with CTLA-4 eradicates established HPV + tumors. When the tumor volume is about 50mm^3^, TC-1 tumor bearing mice are divided into groups treated with CCL11-E6E7 vaccination with or without anti-PD-1, anti PD-L1, anti-CTLA-4 administration every three days for a total of 6 times. **A** Tumor growth curve of plasmid vector control (Vehicle) and anti PD-1, anti PD-L1, anti-CTLA-4 administration alone, data were presented with mean ± SEM (*n* = 3 for vehicle, *n* = 5 for other groups). **B**-**E** kinetics of tumor growth was shown for individual mice of CCL11-E6E7 vaccination with or without anti PD-1, anti PD-L1, anti-CTLA-4 administration. (F) Flow cytometry analysis of E7 specific CD8 + T cell levels in each group on the 14th day after CCL11-E6E7 immunization data were presented with mean ± SEM (*n* = 3 for vehicle, *n* = 5 for other groups). **G** The different chemokines could shape antigen-induced immune types which are summarized in the diagram

As 25 µg CCL11-E6E7 vaccine monotherapy was sufficient to eliminate the subcutaneous TC-1 HPV16 E6E7 + tumor (Fig. 1C, Fig. S1C, Fig. 4B and G), we decreased the CCL11-E6E7 vaccine dose to 10 µg to evaluate the combination effect. After the TC-1 tumors reached an average size of 50 mm^3^, CCL11-E6E7 immunization was given with or without concomitant PD-1, PD-L1 and CTLA-4 checkpoint inhibitors. All three checkpoint inhibitors were administered intraperitoneally every three days, and only the anti-PD-L1 antibody showed early monotherapy-mediated tumor growth inhibition (Fig. 6A). Furthermore, the combination of the anti-CTLA-4 treatment with vaccination induced complete tumor regression in 3/5 mice (Fig. 6B-E), and PD-L1 blockade combined with the vaccination promoted tumor regression in 2/5 mice (Fig. 6B-E), compared to only 1/5 mice achieving tumor regression with CCL11-E6E7 immunization alone. We also analyzed E7-specific CD8 + T cells and found that compared to the 25 µg dose, 10 µg CCL11-E6E7 alone caused a significant decrease in the level of E7-specific CD8 + T cells, which was significantly upregulated by anti-CTLA-4 treatment. PD-1 blockade had no effect on the changes in E7-specific CD8 + T-cell levels induced by the vaccine. Given that anti-PD-L1 monotherapy had a certain inhibitory effect, our results suggest that combination with CTLA-4 blockade is more appropriate for CCL11-E6E7 DNA vaccines.

## Discussion

Although there has been some progress in tumor therapeutic nucleic acid vaccines, clinical experimental results have shown limited efficacy. To further improve the immunogenicity of antigens delivered by nucleic acid vaccines has become an urgent problem to be solved. In this study, we systematically evaluated the effect of chemokine-fused antigens on antigen immunogenicity. The results showed that there were differences in the ability of different chemokines to induce cellular and humoral immunity as summarized in Fig. 6G. Among them, CCL11 had the strongest capacity to induce cellular immunity. CX3CL1 induced the strongest humoral immune response. Both types of immune responses were generated by CXCL6, CXCL9, CXCL10, CXCL14 and CCL14. We further investigated whether CCL11 could enhance antitumor activity and found that CCL11-E6E7 had potent antitumor effects. In general, large tumors are difficult to be eradicated with therapeutic DNA vaccines in tumor animal models. This is due to the rapid tumor growth and DNA-induced immune responses requiring an onset time lasting more than ten days. However, the E6/E7 DNA vaccine fused to CCL11 could eliminate large established HPV16 E6/E7 tumors in multiple repeated experiments, confirming CCL11 as a powerful enhancer of immunogenicity. Further, this strategy was also found to be suitable for mRNA vaccine development, expanding the use scenarios of the future. CCL11-E6E7 induced infiltration of innate and adaptive immune cell subpopulations into tumors and generated significantly diverse T-cell clonal subpopulations within the tumor. CCL11-E6E7 also increased the number of stem-like CD8 + T cells, which are believed to be the cells responsible for response to immune checkpoint therapy, in lymph nodes. The antitumor activity of CCL11-E6E7 was enhanced by anti-CTLA-4 treatment, indicating a synergy produced by complementary mechanisms in promoting cellular immune responses. The current mechanistic studies revealed that the antitumor effects of the CCL11-E6E7 DNA vaccine are partially dependent on CCR3-positive cells, though the exact subset of CCR3-positive cells and the mechanism through which they induce these effects need to be further identified.

In the design of antigens for vaccine development, many pathogen protein sequences, such as those of diphtheria or tetanus toxins, have been incorporated to increase immunogenicity [7]. Although immune-enhancing effects have been observed in mice, such approaches can be counterproductive because significant differences in the immunogenicity of antigens may offset the immune responses. The main response generated by high affinity antigens will suppress responses against insufficient MHC affinity antigens. The present strategy of fusion with chemokines can avoid this undesirable outcome, as chemokines are self-antigens and will not cause a response.

Antigen fused with CCL11 would artificially introduce the expression of CCL11, which may produce short-term or long-term pathophysiological effects through its receptor expressing cells. This requires a comprehensive analysis based on the pharmacokinetic characteristics of plasmid DNA and the tissue distribution patterns. Previous studies have extensively investigated the pharmacokinetic characteristics of plasmid DNA vaccines. The plasmids would be completely eliminated in the vast majority of organs within 14 days after injection. Even at the injection site, it does not exceed 28 days which is also applicable for the expressed antigens [31, 32]. Therefore, CCL11-E6E7 would also show short-term expression in animals and cause no long-term effects on normal animal cells or CCR3 + cells in particular. CCL11 is a member of the eotaxin family which are chemotactic agents for eosinophils and participate innate immunity. CCL11 can selectively recruit eosinophils into inflammatory sites. In the inflammatory sites, T-helper 2 cytokines, such as interleukin-4 (IL-4) and IL-10 induce eosinophils, T cells, B cells and macrophages to produce CCL11. CCR3, the main receptor of CCL11 is expressed on mast cells, eosinophils, Th2 lymphocytes, and keratinocytes which are the effect cells for tissue inflammation [33]. Therefore, CCL11 is mainly produced by the inflammatory site and promotes local inflammation acting as an effect factor rather than a prime factor. Our plasmid DNA is mainly translated in muscles, and local inflammation occurs at the injection site whose short-term effects are unlikely to cause chronic allergic diseases such as asthma. However, to apply CCL11 in vaccine preparation, systematic and comprehensive safety evaluations are still needed. Moreover, CCL11 may also regulate eosinophil migration in the tumor microenvironment through its interaction with CCR3, and its significant role has been confirmed in colorectal cancer, Hodgkin lymphoma, and oral squamous cell carcinoma [34, 35]. Whether eosinophils function in tumor promotion or tumor elimination is still unclear. The antitumorigenic role of eosinophils has been well described in several studies [36, 37]. Adoptive transfer of eosinophils into mice promoted lung metastasis in multiple tumor models [38]. Our results suggest the involvement of eosinophils in the immunogenic enhancement caused by vaccination and the potential of targeting eosinophils for vaccine applications. However, due to the broad expression of CCR3 and the receptor diversity of CCL11, the exact role of eosinophils in vaccination still needs to be investigated, by using multiple gene knockout mouse models, which is a limitation of our study.

In the investigation of which specific immune cell subpopulations influence the therapeutic efficacy of the CCL11-E6E7 DNA vaccine, we found that CD8 + T cells play a critical role, which is consistent with their role in other types of vaccines. However, the removal of CD4 + T cells also resulted in a decrease in specific CD8 + T-cell levels. Although significant antitumor activity was maintained, the rate of tumor clearance was reduced, indicating both types of cells are needed for the complete eradication of tumors. Intriguingly, NK cells depletion not only reduced the specific CD8 + T-cell level but also decreased the tumor eradication rate. This result highlights the important role of NK cells in the recruitment of cDC1s into the tumor microenvironment for the initiation of the antitumor response [39].

Multiple types of tumor immunotherapy are recommended to be combined to successfully activate the cancer-immunity cycle and overcome tumor immune escape and immunosuppressive effects [40]. Thus, many tumor therapeutic vaccines are being combined with immune checkpoint therapy. We have explored the combined effects of multiple immune checkpoints with our DNA vaccines. In which, a combination of CCL11-E6E7 DNA vaccine with anti-CTLA-4 exhibited the greatest treatment benefit, [41]. As an immunosuppressive checkpoint, targeting CTLA-4 to activate immunity for cancer treatment has been widely studied. Two CTLA-4 blocking monoclonal antibodies, ipilimumab (IgG1) and tremelimumab (IgG2), are clinically approved and have shown efficacy in a subset of solid tumor patients [42, 43]. Since CTLA-4 competes with the costimulatory receptor CD28 for the CD80 and CD86 ligands to suppress T cell activation, the removal of CTLA-4-mediated negative co-stimulation to augment effector T-cell-mediated immune responses has been identified as the central mechanism of anti-CTLA-4 [44]. In addition, a high level of CTLA-4 is expressed on the surface of regulatory T cells (Tregs) [45]. Several murine studies have suggested CTLA-4 blockade may impair the suppressive activity of Tregs and deplete Tregs within the tumor microenvironment (TME) via antibody-dependent cell-mediated cytotoxicity (ADCC) resulting in reduced tumor immunosuppression and expansion of effector T cells [46–48]. In fact, a previous study found that peptide vaccines combined with anti-CTLA-4 significantly reduced the proportion of intratumoral Tregs in the same TC-1 tumor model [41]. In our study, we observed an increased number of E7 specific effector T cells in peripheral blood (Fig. 6F). Therefore, it is possible that both effects of CTLA-4 blockade played a role in enhancing the efficacy of DNA vaccines, although DNA vaccine treatment did not significantly increase the proportion of Tregs in tumors. Several vaccines have shown the effectiveness of combining PD-1 [49]. PD-1 functions by reversing exhausted T cells. In our study, to determine the reason why the combination of the CCL11-E6E7 vaccine and PD-1 is not effective, further tests are needed to determine whether the tumor specific T cells induced by DNA vaccines are in a PD-1-responsive state. We will further evaluate whether anti-CTLA-4 also reduces the proportion of Tregs and the state of E6E7 specific T cells in tumors in future studies. Therefore, we encourage experimentally combining various immune checkpoint therapies with different vaccine platforms, to identify potential pharmacologically synergistic combinations, rather than simply superimposing the efficacy of two separate drug therapies.

In our study, electroporation was used for plasmid delivery. Electroporation has been demonstrated an adjuvant-like property for plasmid DNA delivery. Electroporation in skeletal muscle would release a danger signal to recruit antigen-presenting cells independently of plasmid DNA administration [50]. The DNA vaccine delivered by electroporation showed levels of antibodies that were equivalent to those of cationic lipids and a stronger T cell immune response [51]. The preventive COVID-19 vaccine and the therapeutic HPV vaccine delivered by electroporation have finished Phase II clinical trials and shown good safety [1, 52]. However, it is indeed uncomfortable and painful which may lead to a poor compliance. Due to the convenience of use, there are difficulties in popularizing electroporation, especially when using preventive vaccines that are targeted to healthy individuals. In future applications, we note that needle-free injection can be used as an alternative solution, such as the Indian DNA vaccine approved for COVID-19 [31].

## Conclusions

### Main conclusions of the research

We have unprecedentedly fused and expressed all potential chemokines with HPV16 E6/E7 antigens and systematically analyzed chemokines that could enhance the immunogenicity of antigens, from which several chemokines that can significantly enhance the anti-tumor activity of antigens were identified. Among them, CCL11 showed the strongest anti-tumor enhancing effect, as evidenced by a high percentage of clearance of the large established tumors in multiple repeated experiments which was not shown by previous DNA vaccines. The fusion antigen with CCL11 not only increased the number of specific CD8 + T cells, but also improved the quality of the T cells, as manifested by an increase in TCF1 + PD1- CD62L + stem-like CD8 + T cells and clonal diversity. Simultaneously, a comprehensive tumor-lethal microenvironment with both innate and specific immune cells was induced in the tumor. Mechanistic studies have shown that CCR3 + cells are involved in these immune-enhancing effects. While further identification of specific CCR3 + subpopulations is needed, it also suggests that the involvement of a diversity of cell subpopulations is required for effective antitumor vaccination.

### Importance and relevance

Our study identified candidates for improving the immunogenicity of nucleic acid vaccines and provided new insights into the possible mechanisms of vaccine function.

### Supplementary Information

**Supplementary Material 1.**

**Supplementary Material 2.**

**Supplementary Material 3.**

**Supplementary Material 4.**

**Supplementary Material 5.**

## Data Availability

The datasets used and/or analysed during the current study are available from the corresponding author upon reasonable request.

## Abbreviations

MDSC: Myeloid-derived suppressor cells
OVA: Ovalbumin
NK: Natural killer
DC: Dendritic cell
CIN: Cervical intraepithelial neoplasia
MITD: MHC class I trafficking signals
APCs: Antigen-presenting cells
GAPDH: Glyceraldehyde-3-phosphate dehydrogenase
GO: Gene ontology
KEGG: Kyoto encyclopedia of genes and genomes
GSEA: Gene set enrichment analysis
MPECs: Memory-predictive effector cells
SLECs: Short-lived effector cells
Tcm: Central memory T
DEGs: Differentially expressed genes
HPV: Human papillomavirus
TCF1: Transcription factor 1
PCA: Principal component analysis
mAbs: Monoclonal antibodies
Tregs: Regulatory T cells
TME: Tumor Microenvironment
ADCC: Antibody-dependent cell-mediated cytotoxicity
Lipoplex: (LPX)
IVT: In vitro transcription
