# Supplementary figures and images for: Genetic fusion of CCL11 to antigens enhances antigenicity in nucleic acid vaccines and eradicates tumor mass through optimizing T-cell response
Source: Mol Cancer. 2024 Mar 8;23:46. doi: 10.1186/s12943-024-01958-4 (PMC10921619; doi:10.1186/s12943-024-01958-4)

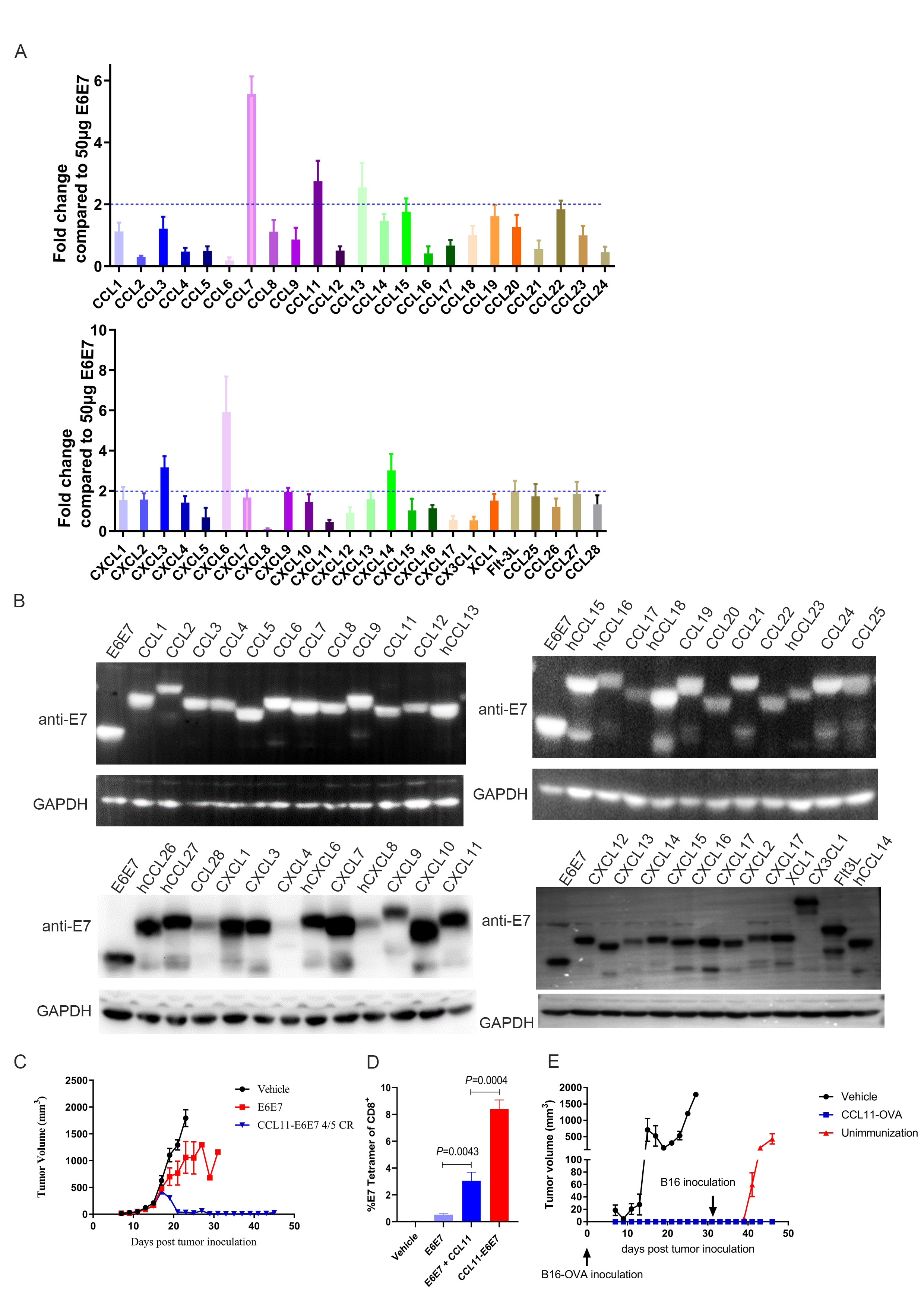

Supplement: Supplementary file 1 — Supplementary Material 1. [file 12943_2024_1958_MOESM1_ESM.jpg]

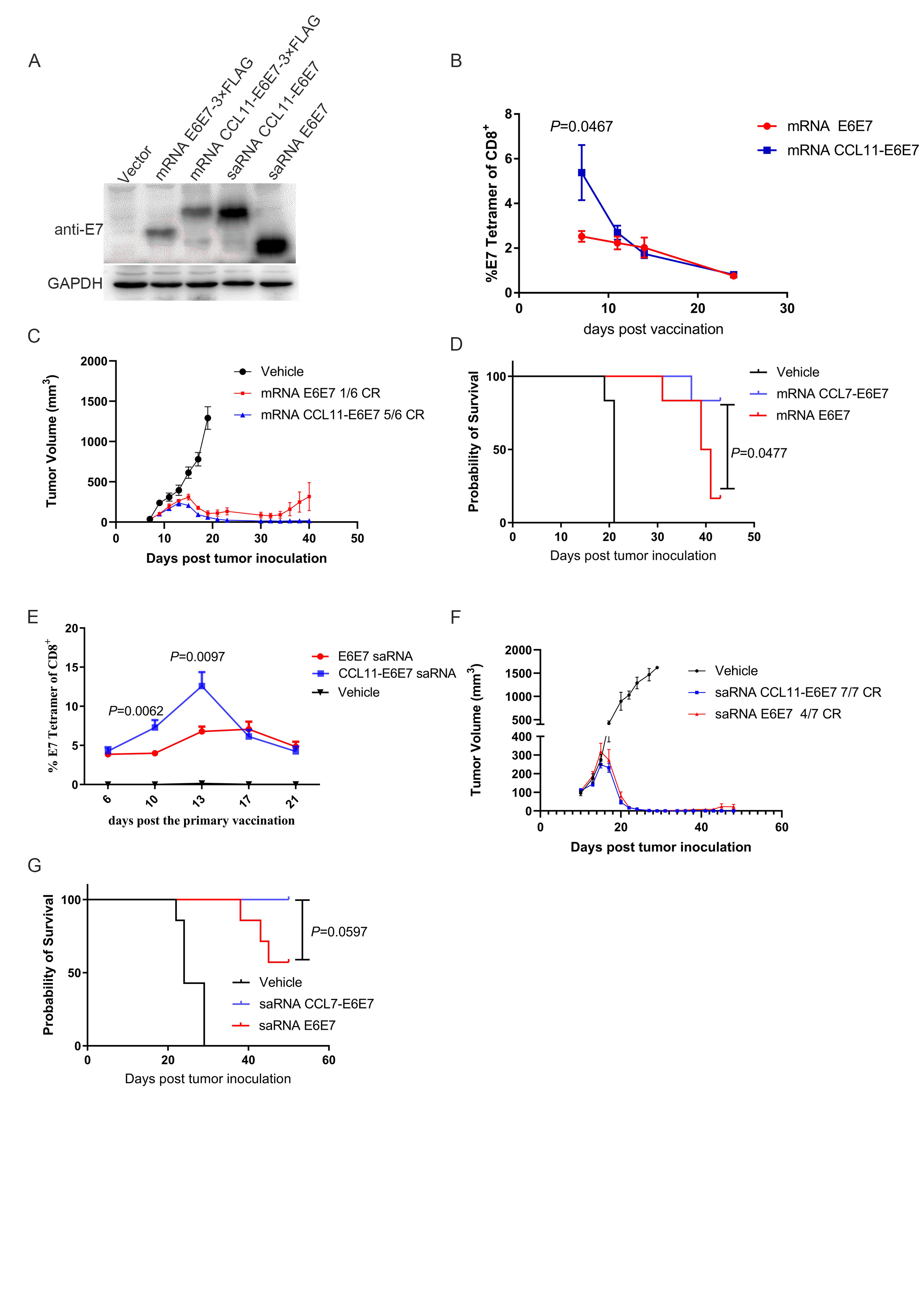

Supplement: Supplementary file 2 — Supplementary Material 2. [file 12943_2024_1958_MOESM2_ESM.jpg]

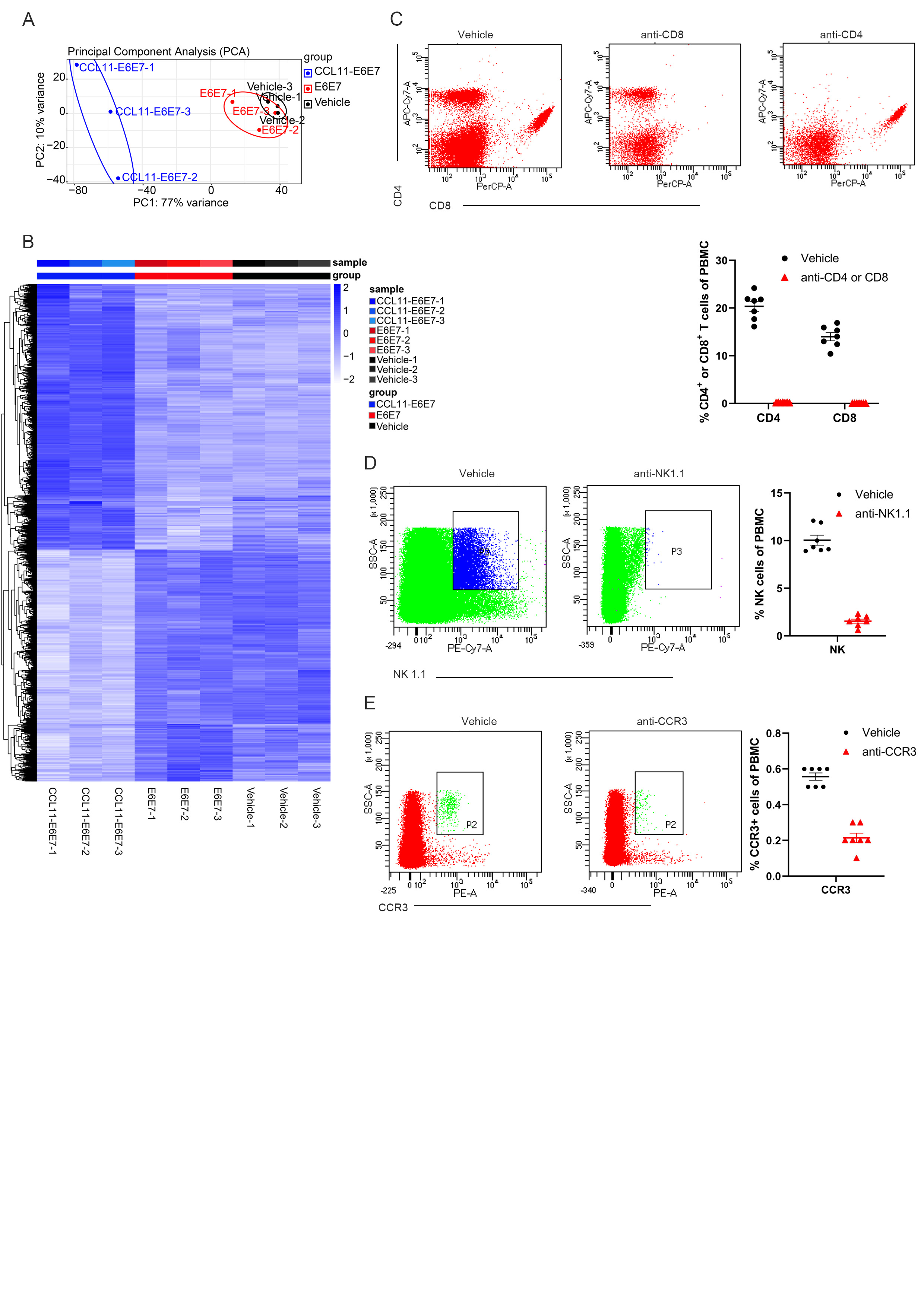

Supplement: Supplementary file 3 — Supplementary Material 3. [file 12943_2024_1958_MOESM3_ESM.jpg]
